# Maternal and neonatal outcomes in pregnancies complicated with pulmonary hypertension: a retrospective cohort study

**DOI:** 10.1186/s40001-025-02987-5

**Published:** 2025-08-06

**Authors:** Gezi Chen, Zhenyu Zhang, Xianlan Zhao, Kai Huang

**Affiliations:** 1 Department of Obstetrics, The First Affiliated Hospital of Zhengzhou University, Zhengzhou, Henan China; 2 Center for Reproductive Medicine, The First Affiliated Hospital of Zhengzhou University, Zhengzhou, Henan China

**Keywords:** Pulmonary hypertension (PH), Pregnancy, Maternal outcome, Neonatal outcome, Prognosis

## Abstract

**Background:**

To evaluate the maternal and neonatal outcomes of pregnancy complicated with pulmonary hypertension.

**Methods:**

We reviewed and analyzed the clinical data of 70 pregnant women with PH admitted to the First Affiliated Hospital of Zhengzhou University from January 2012 to December 2022. The data extracted included general characteristics, laboratory findings, imaging manifestations, and maternal and perinatal outcomes. PH was diagnosed via echocardiography, and patients were categorized into mild and moderate-to-severe PH groups on the basis of pulmonary artery systolic pressure (sPAP). Cardiac function was graded according to the World Health Organization Functional Class (WHO-FC).

**Results:**

The mean age of the patients was 29.83 ± 5.81 years. A total of 55.71% were primigravida, and 44.29% were multigravida. The majority of patients (65.71%) had regular obstetric examinations during pregnancy. There were statistically significant differences in BNP levels, right ventricular diastolic diameter (RVDD), and WHO-FC between the mild and moderate-to-severe PH groups. The majority of patients (87.50% vs. 86.67%) underwent cesarean section, with regional anesthesia used in 70.00% of the patients. The maternal mortality rate was 5.71% (4/70) in women with severe PH. The median gestational age at delivery was 36.57 weeks for mild PH group and 33.93 weeks for moderate-to-severe PH group, respectively. Fetal birth weight was significantly lower in the moderate-to-severe PH group. A greater proportion of infants in the moderate-to-severe PH group were admitted to the neonatal intensive care unit (NICU) and had low birth weights (LBWs).

**Conclusions:**

Pregnancy complicated with PH poses a significant risk to both maternal and neonatal health, with poorer outcomes associated with moderate-to-severe PH. Multidisciplinary management, early detection, and timely intervention are crucial for improving outcomes. This study underscores the importance of individualized care plans and the need for further research to refine the predictive factors for the prognosis of PH-complicated pregnancies.

## Background

Pulmonary hypertension (PH) is a serious clinical syndrome defined by a mean pulmonary artery pressure (mPAP) ≥ 25 mmHg at rest. To date, the gold standard for diagnosing PH is right heart catheterization. However, the procedure is not feasible for routine clinical examinations because of its invasive nature. Echocardiography is commonly used as an alternative diagnostic approach. The prevalence rate of PH is reported to be 9.7 per 100,000, with a female-to-male ratio of 1.8:1. Women of reproductive age constitute the majority of female patients. Moreover, due to impaired pulmonary vascular distensibility and unique physiological alterations during pregnancy, the prognosis of pregnant women with PH is poor [1, 2].

At present, PH is considered a contraindication for pregnancy owing to high maternal and fetal morbidity and mortality [3]. Studies have reported that the maternal mortality rate of pregnant patients with PH is approximately 30–56% [4]. With recent therapeutic advances, maternal mortality during pregnancy has remained high at 15.8% [5]. The causes of maternal death reported include pulmonary hypertension crisis, right heart failure, pulmonary disease and embolism, whereas adverse fetal outcomes include induced abortion, intrauterine death, premature delivery, and fetal growth restriction. Early recognition of PH poses challenges, as initial clinical manifestations are often atypical and difficult to distinguish from pregnancy status. Echocardiography serves as a screening method for high-risk populations. Upon the diagnosis of pulmonary hypertension, an emphasis should be placed on individualized management and multidisciplinary collaboration. The timing of pregnancy termination, mode of delivery, and anesthesia method should be determined on the basis of maternal and fetal status. Although pregnancy is contraindicated for patients with pulmonary hypertension, certain female patients actively plan pregnancies, while others are diagnosed with PH during pregnancy [6].

Early detection and prompt treatment significantly reduce maternal and fetal mortality associated with PH. However, in low-income and middle-income countries, patients frequently present for medical care only after complications have occurred [7]. Data regarding this special population remain limited. Therefore, we contribute our findings on managing pregnant women with PH, thereby augmenting the literature.

## Methods

### General data

The study included patients with PH who were admitted to the First Affiliated Hospital of Zhengzhou University, the largest tertiary center in China, from January 2012 to December 2022. Approval for the study was obtained from the institutional review board at our hospital. The need for informed consent was waived by the Ethics Committee because the study was an observational, retrospective study using a database from which the patients’ identification information was removed. Data extracted from clinical medical records included general characteristics, laboratory findings, imaging manifestations, and maternal and perinatal outcomes.

### PH diagnosis and cardiac function classification

Diagnostic criteria for pregnancy complicated with PH: PH refers to an elevation in pulmonary artery pressure caused by various factors, and the mean pulmonary artery pressure (mPAP) detected by right heart catheterization in the resting state at sea level ≥ 25 mmHg (1 mmHg = 0. 133 kPa) [8]. Owing to the invasive nature of right heart catheterization, stringent technical personnel requirements, and potential radiation risk to the fetus, echocardiography can be used for initial diagnosis during pregnancy. PH can be categorized into three levels according to the pulmonary artery systolic pressure (sPAP) detected by echocardiography: severe PH (≥ 80 mmHg), moderate PH (50–79 mmHg) and mild PH (30–49 mmHg) [3]. Moderate and severe PH were combined in the study for the following reasons: (a) The limited number of severe PH cases undermined the statistical power; (b) The clinical management pathways were similar according to the guidelines [3, 20]; (c) The mortality risk profiles were comparable [16, 22]. Cardiac function was graded according to the World Health Organization Functional Class (WHO-FC), which was divided into grades I-IV to evaluate heart status. Women with PH are also classified into three subgroups: congenital heart disease (CHD-PAH), idiopathic disease (iPAH), and other PH (oPAH).

### Statistical analysis

All the statistical analyses were conducted via IBM SPSS Statistics for Windows, version 24.0. Descriptive statistics are expressed as the means with SDs (±) or medians with interquartile ranges (IQRs) for continuous variables and as counts and proportions for categorical variables. Comparisons between groups were assessed via Student’s *t* test, the Mann–Whitney U test, or the chi-square test, as appropriate. *p* < 0.05 was used to indicate statistical significance.

## Results

A total of 70 pregnant women with PH were included in this study. The general characteristics of the patients are summarized in Table 1. The mean age of the patients was 29.83 ± 5.81 years. Among the patients, 55.71% were primigravida, and 44.29% were multigravida. A total of 46 women (65.71%) had regular obstetric examinations during pregnancy. Patients were further divided into mild PH (N = 40, median sPAP 37.00 mmHg [IQR, 34.75–42.25]) and moderate-to-severe PH (N = 30, median sPAP 74.50 mmHg [IQR, 56.0–96.5]). The median gestational week was 36.22 (IQR, 34.00–38.11) and 32.07 (IQR, 24.79–36.24) weeks for mild PH and moderate-to-severe PH, respectively. The vast majority of women with PH had a normal ejection fraction (EF) (62.00%, [IQR, 60.00–63.00]) and left ventricular end-diastolic diameter (LVDD) (43.00 mm, [IQR, 39.25–48.00]), regardless of the severity of PH. The RVDD increased in the moderate-to-severe PH group (26.50 vs. 18.00, P < 0.001). Compared with those in the mild PH group, women with moderate-to-severe PH had higher BNP levels (430.50 vs. 142.98, P = 0.013) and poorer NYHA functional classes (40.00% vs. 15.00%, P = 0.018). Among the 70 women with PH, bidirectional shunts occurred in 6 of the 30 women in the moderate-to-severe PH group (20.00%), whereas no bidirectional shunts occurred in the mild PH group. In terms of PH classification, oPAH was most common (42.50%, 17/40) in the mild PH group, whereas CHD-PAH was the most common (66.67%, 20/30) in the moderate-to-severe PH group. Maternal mortality occurred in 0 and 4 (13.33%) women with mild or moderate-to-severe PH, respectively.

**Table 1:** Baseline characteristics of the participants

Variables	Total (n = 70)	Mild PH (n = 40)	Moderate to severe PH (n = 30)	*P* value
Age, mean ± SD	29.83 ± 5.81	29.98 ± 5.52	29.63 ± 6.26	0.81
Gestational weeks, Median (IQR)	35.29 (29.85, 37.79)	36.22 (34.00, 38.11)	32.07 (24.79, 36.46)	0.008
Gravida, n (%)				0.89
Primigravida	39 (55.71%)	22 (55.00%)	17 (56.67%)	
Multigravida	31 (44.29%)	18 (45.00%)	13 (43.33%)	
Regular obstetric examinations, n (%)	46 (65.71%)	28 (70.00%)	18 (60.00%)	0.383
sPAP, mmHg, Median (IQR)	47.50 (36.25, 70.00)	37.00 (34.75, 42.25)	74.50 (61.00, 99.00)	< 0.001
EF, %, Median (IQR)	62.00 (60.00, 63.00)	62.00 (60.00, 63.00)	62.00 (60.00, 64.75)	0.895
LVDD, mm, Median (IQR)	43.00 (39.25, 48.00)	43.00 (40.00, 48.25)	44.00 (36.25, 48.00)	0.721
RVDD, mm, Median (IQR)	21.00 (17.00, 26.00)	18.00 (15.75, 22.00)	26.50 (21.25, 32.00)	< 0.001
BNP, pg/ml, Median (IQR)	207.95 (75.28, 907.73)	142.98 (58.00, 517.17)	430.50 (125.68, 1829.00)	0.013
Bi-directional shunt, n (%)	6 (8.57%)	0 (0.00%)	6 (20.00%)	0.012
NYHA, n (%)				0.018
I–II	52 (74.29%)	34 (85.00%)	18 (60.00%)	
III–IV	18 (25.71%)	6 (15.00%)	12 (40.00%)	
Classification of PH, n (%)				0.025
CHD-PAH	35 (50.00%)	15 (37.50%)	20 (66.67%)	
iPAH	9 (12.86%)	8 (20.00%)	1 (3.33%)	
oPAH	26 (37.14%)	17 (42.50%)	9 (30.00%)	
Maternal death, n (%)	4 (5.71%)	0 (0.00%)	4 (13.33%)	0.063

The management of pregnancies with PH is detailed in Table 2. The proportion of cesarean Sects. (87.50% vs. 86.67%) was significantly greater than that of vaginal deliveries (12.50% vs. 13.33%) in both groups. The regional anesthesia rate and general anesthesia rate were 70.00% (49/70) and 24.29% (17/70), respectively. The pregnancy was terminated in 13 of 70 patients (18.57%) at an early stage (< 24 weeks). A total of 86.67% (26/30) of the pregnant women with moderate-to-severe PH were transferred to the ICU after delivery, while the proportion in the mild PH group was 7.50% (3/40). PH-targeted therapy was given to 11 women (15.71%, 11/70) with moderate-to-severe PH. Extracorporeal membrane oxygenation (ECMO) was administered to one woman with multiple organ dysfunction syndrome (MODS), but it did not save her life.

**Table 2:** Management in pregnancies with PH

Variables	Total (n = 70)	Mild PH (n = 40)	Moderate to severe PH (n = 30)	*P* value
Mode of delivery, n (%)				1.000
Vaginal	9 (12.86%)	5 (12.50%)	4 (13.33%)	
Cesarean section	61 (87.14%)	35 (87.50%)	26 (86.67%)	
Anesthesia method, n (%)				0.311
Regional anesthesia	49 (70.00%)	30 (75.00%)	19 (63.33%)	
General anesthesia	17 (24.29%)	7 (17.50%)	10 (33.33%)	
None	4 (5.71%)	3 (7.50%)	1 (3.33%)	
Termination of pregnancy, n (%)	13 (18.57%)	5 (12.50%)	8 (26.67%)	0.272
Transferred to ICU, n (%)	29 (41.43%)	3 (7.50%)	26 (86.67%)	< 0.001
Targeted therapy, n (%)	11 (15.71%)	0 (0.00%)	11 (36.67%)	< 0.001
ECMO, n (%)	1 (1.43%)	0 (0.00%)	1 (3.33%)	0.429

Table 3 presents the fetal and neonatal outcomes. The median gestational week at delivery was 36.57 (IQR, 35.29–38.57) weeks for the mild PH group and 33.93 (IQR, 31.78–37.11) weeks for the moderate-to-severe PH group. Among the 57 infants born to pregnant patients with pulmonary hypertension, 33 (57.89%) were preterm (gestational age < 37 weeks). The birth weights were 2750.00 (IQR, 2425.00–3200.00) g for mild PH cases and 2280.00 (IQR, 1410.00–2787.50) g for moderate-to-severe PH cases. In the mild PH group, 8 infants (22.86%) had low birth weights (LBWs), while in the moderate-to-severe PH group, 11 infants (50.00%) had LBWs. A total of 30 infants (52.63%) were admitted to the neonatal intensive care unit (NICU), with a median length of hospital stay of 12.50 (IQR, 6.00–28.75) days. Two (3.51%) neonates died as a result of prematurity. The incidence of neonatal asphyxia was 12.28% (7/57).

**Table 3:** Fetal and neonatal outcome

Variables	Total (n = 57)	Mild PH (n = 35)	Moderate to severe PH (n = 22)	*P* value
Gestational weeks, Median (IQR)	36.14 (33.57, 38.00)	36.57 (35.29, 38.57)	33.93 (31.78, 37.11)	0.029
Preterm delivery (< 37 wk), n (%)	33 (57.89%)	18 (51.43%)	15 (68.18%)	0.212
Birth weight, Median (IQR)	2650.00 (1900.00, 3050.00)	2750.00 (2425.00, 3200.00)	2280.00 (1410.00, 2787.50)	0.037
Low birth weight, n (%)	19 (33.33%)	8 (22.86%)	11 (50.00%)	0.034
Transferred to NICU, n (%)	30 (52.63%)	15 (42.86%)	15 (68.18%)	0.062
NICU stay (d), Median (IQR)	12.50 (6.00, 28.75)	9.00 (6.50, 17.00)	15.00 (5.50, 30.50)	0.618
Offspring death, n (%)	2 (3.51%)	0 (0.00%)	2 (9.09%)	0.145
Neonatal asphyxia, n (%)	7 (12.28%)	4 (11.43%)	3 (13.64%)	1.000

## Discussion

Pulmonary hypertension is considered a contraindication for pregnancy. The physiological changes that occur during pregnancy lead to an approximately 50% increase in plasma volume and cardiac output, which peak at 32–34 weeks of gestation. During delivery, the cardiac output may increase by an additional 30–50%, leading to an overall increase of 80% [9]. These alterations are well tolerated during pregnancy and the postpartum period in healthy women. In pregnant women with underlying diseases such as congenital heart disease, hypertension disorders, and connective tissue disease, these changes may result in serious complications, including cardiopulmonary failure, arrhythmia, pneumonia, pulmonary embolism, venous thromboembolism, pulmonary interstitial fibrosis, and even death. Sliwa et al. reported that the maternal mortality rate during pregnancy complicated by pulmonary hypertension ranges from 30 to 56%, whereas the perinatal mortality rate is as high as 11–28% [10].

In this study, a cohort of 70 pregnant women with PH were enrolled. Congenital heart disease (CHD) was found to be the most prevalent cause of PH during pregnancy, which is consistent with prior literature [11]. The results revealed statistically significant differences in certain indices, such as BNP, RVDD, and sPAP, between the two groups. BNP, pro-BNP, and N-terminal pro-BNP are effective indicators of prognosis in patients with heart failure, whether symptomatic or not [12]. MiR-21 and BNP are highly expressed in patients with preeclampsia complicated with heart failure and are expected to be important biomarkers for diagnosing preeclampsia complicated with heart failure and assessing the degree of heart failure in these patients, as reported by Kan’s study [13]. Statistically significant disparities in the World Health Organization functional class (WHO-FC) were detected between the mild PH group and the moderate-to-severe PH group. Patients with poorer cardiac function were more likely to have a poor prognosis. Further research with large datasets should integrate novel biomarkers (e.g., NT-proBNP, miR-21) with hemodynamic parameters to refine risk stratification.

As noted by Weiss, the 1-month postpartum period is a high-risk period for death in patients with PH [14]. In our cohort, 4 patients, all of whom had severe PH, died within 2 weeks after delivery, representing a mortality rate of 5.71% (4/70), which is lower than that in previous reports (17–33%) [14–16]. There is a lack of consensus or standard guidelines on the optimal timing and method for terminating pregnancies in patients with PH. A multidisciplinary team (MDT) consisting of obstetricians, cardiologists, neonatologists, and anesthesiologists should carefully evaluate the patient’s pulmonary artery pressure, cardiac function, fetal intrauterine status, and gestational age to determine the appropriate termination time and method [17, 18]. In a study by Nadine et al., it was observed that women with well-controlled PH who received close monitoring by a multidisciplinary team had positive outcomes during and after pregnancy with successful deliveries of healthy newborns [19]. Timely admission to a tertiary care center, early assessment by a multidisciplinary team, and strict perinatal supervision may contribute to favorable outcomes.

Extensive application of cesarean section under regional anesthesia may be another potential contributor to improved outcomes. The majority of the study participants (81.43%, 57/70) elected to continue their pregnancies, whereas 18.57% (13/70) opted for termination of pregnancy. Negative pressure suction is recommended as the preferred termination method for pregnant women with PH in early pregnancy, whereas after 13 weeks of gestation, cesarean section is recommended for severe PH or grade III-IV cardiac function or serious PH complications [20]. However, controversy remains regarding how to terminate pregnancy in pregnant women with mild to moderate PH and grade I-II cardiac function [16, 21]. Cesarean section is currently preferred to vaginal delivery for terminating pregnancy in patients with PH, as it can shorten the second stage of labor and reduce acidosis, hypercapnia, and hypoxia resulting from prolonged delivery [22, 23]. This study revealed that 87.14% of pregnant patients with PH underwent cesarean section, with most experiencing favorable outcomes. Studies have demonstrated that, compared with general anesthesia, spinal anesthesia can prevent abrupt changes in hemodynamics and reduce the duration of postoperative mechanical ventilation, the length of stay in the cardiac intensive care unit, and the total hospital stay [24].

During cesarean section, close monitoring of central venous pressure, invasive arterial pressure, oxygen saturation, and urine output is essential. The regulation of fluid infusion is critical for preventing heart failure, and low-molecular-weight heparin should be promptly administered postdelivery to prevent venous thrombosis [20]. In high-risk patients, ECMO preparations should be made in advance to manage postpartum heart failure. However, given the financial constraints in many countries, access to ECMO may be limited. PH medications should be continued during pregnancy, except for endothelin receptor antagonists such as ambrisentan, which should be discontinued upon pregnancy confirmation and reintroduced postdelivery [25]. In terms of fetal and neonatal outcomes, the incidences of premature delivery (57.89%) and LBW (33.33%) were elevated in pregnant women with PH, particularly in the moderate-to-severe PH group (68.18% and 50.00%, respectively).

In our study, patients mostly presented to the hospital during the last trimester or upon symptom occurrence, resulting in a median gestational age at delivery of 36.14 (IQR, 33.57–38.00) weeks. This could be attributed to a lack of awareness regarding the increased risks associated with continuing pregnancy in patients with PH. Enhancing social health education by relevant departments is essential to increase disease awareness, particularly in low socioeconomic and culturally underprivileged communities. This approach aims to raise awareness about the severity of the disease and the critical importance of timely treatment and surgical intervention before pregnancy, which can improve pregnancy outcomes and reduce cardiovascular complications.

This research offers valuable real-world perspectives on the management and outcomes of pregnancy complicated by pulmonary hypertension, a high-risk condition with scarce contemporary data. Its primary advantages encompass a relatively large cohort size (n = 70) for this rare condition, a detailed characterization of maternal hemodynamics (BNP, RVDD, sPAP, WHO-FC), and a comprehensive report of both maternal and neonatal outcomes stratified according to PH severity. The focus on practical management aspects (e.g., mode of delivery, choice of anesthesia, use of ECMO) and the emphasis on multidisciplinary care enhance its clinical relevance.

Nonetheless, this study has notable limitations inherent in its retrospective, single—center design, such as potential selection bias, incomplete data acquisition, and the lack of standardized follow-up. The small sizes of subgroups limit the statistical power to identify robust predictors of mortality. Moreover, the absence of long-term maternal or child outcomes restricts the understanding of the full prognostic implications. The generalizability of the findings may be constrained by the single-center setting and regional healthcare practices. Consequently, subsequent multi-center and large-scale studies ought to be conducted.

## Conclusions

PH-complicated pregnancies pose significant risks to maternal and fetal life. High morbidity and mortality rates among pregnant women with PH necessitate multidisciplinary treatment and management to reduce these rates. Prognosis may be informed by potential predictive factors such as WHO-FC, BNP, RVDD, and sPAP. This study is retrospective in nature and thus has limitations due to incomplete data, which impedes follow-up analysis.

## Data Availability

The datasets used and/or analyzed during the current study are available from the corresponding author upon reasonable request.

## Abbreviations

PH: Pulmonary hypertension
CHD: Congenital heart disease
EF: Ejection fraction
WHO-FC: World Health Organization functional class
LVDD: Left ventricular end-diastolic
RVDD: Right ventricular end-diastolic
PAP: Pulmonary artery pressure
ECMO: Extracorporeal membrane oxygenation

## References

[1] Mocumbi A, Humbert M, Saxena A, Jing Z-C, Sliwa K, Thienemann F, et al. Pulmonary hypertension. Nat Rev Dis Primers. 2024;10: 1. 38177157 10.1038/s41572-023-00486-7

[2] Agrawal A, Bajaj S, Bhagat U, Yesilyaprak A, Chandna S, Arockiam AD, et al. Cardiovascular complications with delivery hospitalizations in patients with pulmonary hypertension: a nationwide study from 2011 to 2020. J Am Heart Assoc. 2024;13: e031632. 38804208 10.1161/JAHA.123.031632 PMC11255631

[3] Galiè N, Humbert M, Vachiery J-L, Gibbs S, Lang I, Torbicki A, et al. 2015 ESC/ERS guidelines for the diagnosis and treatment of pulmonary hypertension: the joint task force for the diagnosis and treatment of pulmonary hypertension of the European Society of Cardiology (ESC) and the European Respiratory Society (ERS): endorsed by: Association for European Pediatric and Congenital Cardiology (AEPC), International Society for Heart and Lung Transplantation (ISHLT). Eur Heart J. 2016;37:67–119. 26320113 10.1093/eurheartj/ehv317

[4] Regitz-Zagrosek V, Roos-Hesselink JW, Bauersachs J, Blomström-Lundqvist C, Cífková R, De Bonis M, et al. 2018 ESC guidelines for the management of cardiovascular diseases during pregnancy. Eur Heart J. 2018;39:3165–241. 30165544 10.1093/eurheartj/ehy340

[5] Luo J, Shi H, Xu L, Su W, Li J. Pregnancy outcomes in patients with pulmonary arterial hypertension: a retrospective study. Medicine (Baltimore). 2020;99: e20285. 32501975 10.1097/MD.0000000000020285 PMC7306336

[6] Chin KM, Santiago-Munoz P. Pregnancy and congenital heart disease-associated pulmonary hypertension: are outcomes improving? Circulation. 2023;147:562–4. 36780392 10.1161/CIRCULATIONAHA.122.063191

[7] Dinarti LK, Hartopo AB, Kusuma AD, Satwiko MG, Hadwiono MR, Pradana AD, et al. The congenital heart disease in adult and pulmonary hypertension (COHARD-PH) registry: a descriptive study from single-center hospital registry of adult congenital heart disease and pulmonary hypertension in Indonesia. BMC Cardiovasc Disord. 2020;20:163. 32264836 10.1186/s12872-020-01434-z PMC7137468

[8] Poch D, Mandel J. Pulmonary hypertension. Ann Intern Med. 2021;174: ITC49–64. 33844574 10.7326/AITC202104200

[9] Savu O, Jurcuţ R, Giuşcă S, van Mieghem T, Gussi I, Popescu BA, et al. Morphological and functional adaptation of the maternal heart during pregnancy. Circ Cardiovasc Imaging. 2012;5:289–97. 22455877 10.1161/CIRCIMAGING.111.970012

[10] Sliwa K, van Hagen IM, Budts W, Swan L, Sinagra G, Caruana M, et al. Pulmonary hypertension and pregnancy outcomes: data from the Registry Of Pregnancy and Cardiac Disease (ROPAC) of the European Society of Cardiology. Eur J Heart Fail. 2016;18:1119–28. 27384461 10.1002/ejhf.594

[11] Li M, Yao Q, Xing A. A clinical analysis of 188 cases of pregnancy complicated with critically heart disease. Zhong Nan Da Xue Xue Bao Yi Xue Ban. 2014;39:1145–50. 25432371 10.11817/j.issn.1672-7347.2014.11.007

[12] Kampman MAM, Balci A, van Veldhuisen DJ, van Dijk APJ, Roos-Hesselink JW, Sollie-Szarynska KM, et al. N-terminal pro-B-type natriuretic peptide predicts cardiovascular complications in pregnant women with congenital heart disease. Eur Heart J. 2014;35:708–15. 24334717 10.1093/eurheartj/eht526

[13] Woo JL, DiLorenzo MP, Rosenzweig E, Pasumarti N, Villeda GV, Berman-Rosenzweig E, et al. Correlation between right ventricular echocardiography measurements and functional capacity in patients with pulmonary arterial hypertension. Tex Heart I J. 2022;49: e217719. 10.14503/THIJ-21-7719 PMC9809095 36350291

[14] Weiss BM, Zemp L, Seifert B, Hess OM. Outcome of pulmonary vascular disease in pregnancy: a systematic overview from 1978 through 1996. J Am Coll Cardiol. 1998;31:1650–7. 9626847 10.1016/s0735-1097(98)00162-4

[15] Bédard E, Dimopoulos K, Gatzoulis MA. Has there been any progress made on pregnancy outcomes among women with pulmonary arterial hypertension? Eur Heart J. 2009;30:256–65. 19147605 10.1093/eurheartj/ehn597

[16] Bonnin M, Mercier FJ, Sitbon O, Roger-Christoph S, Jaïs X, Humbert M, et al. Severe pulmonary hypertension during pregnancy: mode of delivery and anesthetic management of 15 consecutive cases. Anesthesiology. 2005;102:1133–7; (**discussion 5A-6A**). 10.1097/00000542-200506000-00012 15915025

[17] Martin SR, Edwards A. Pulmonary hypertension and pregnancy. Obstet Gynecol. 2019;134:974–87. 31599832 10.1097/AOG.0000000000003549

[18] Ballard W, Dixon B, McEvoy CA, Verma AK. Pulmonary arterial hypertension in pregnancy. Cardiol Clin. 2021;39:109–18. 33222807 10.1016/j.ccl.2020.09.007

[19] Corbach N, Berlier C, Lichtblau M, Schwarz EI, Gautschi F, Groth A, et al. Favorable Pregnancy Outcomes in Women With Well-Controlled Pulmonary Arterial Hypertension. Front Med-lausanne. 2021;8: 689764. 34291063 10.3389/fmed.2021.689764 PMC8287120

[20] American College of Obstetricians and Gynecologists’ Presidential Task Force on Pregnancy and Heart Disease and Committee on Practice Bulletins—Obstetrics. ACOG Practice Bulletin No. 212: Pregnancy and Heart Disease. Obstet Gynecol. 2019;133:e320–56. 10.1097/AOG.0000000000003243 31022123

[21] Zhang Q, Zhu F, Shi G, Hu C, Zhang W, Huang P, et al. Maternal outcomes among pregnant women with congenital heart disease-associated pulmonary hypertension. Circulation. 2023;147:549–61. 36780387 10.1161/CIRCULATIONAHA.122.057987

[22] Liu Y, Li H, Li Y, Zhang J, Gu H, Wang J, et al. Outcomes of pregnancy in women with different types of pulmonary hypertension. BMC Cardiovasc Disord. 2023;23:391. 37558980 10.1186/s12872-023-03423-4 PMC10410774

[23] Lv C, Huang Y, Liao G, Wu L, Chen D, Gao Y. Pregnancy outcomes in women with pulmonary hypertension: a retrospective study in China. Bmc Pregnancy Childb. 2023;23:16. 10.1186/s12884-023-05353-7 PMC9830858 36624418

[24] Rex S, Devroe S. Anesthesia for pregnant women with pulmonary hypertension. Curr Opin Anesthesiol. 2016;29:273–81. 10.1097/ACO.0000000000000310 26978591

[25] Preston IR, Howard LS, Langleben D, Lichtblau M, Pulido T, Souza R, et al. Management of pulmonary hypertension in special conditions. Eur Respir J. 2024;64:2401180. 39209477 10.1183/13993003.01180-2024 PMC11525332

